# Stretchable and durable HD-sEMG electrodes for accurate recognition of swallowing activities on complex epidermal surfaces

**DOI:** 10.1038/s41378-023-00591-3

**Published:** 2023-09-18

**Authors:** Ding Zhang, Zhitao Chen, Longya Xiao, Beichen Zhu, RuoXuan Wu, ChengJian Ou, Yi Ma, Longhan Xie, Hongjie Jiang

**Affiliations:** 1 Shien-Ming Wu School of Intelligent Engineering, South China University of Technology, Guangzhou, 511442 P. R. China; 2 School of Biomedical Sciences and Engineering, South China University of Technology, Guangzhou, 511442 P. R. China

**Keywords:** Electrical and electronic engineering, Materials science

## Abstract

Surface electromyography (sEMG) is widely used in monitoring human health. Nonetheless, it is challenging to capture high-fidelity sEMG recordings in regions with intricate curved surfaces such as the larynx, because regular sEMG electrodes have stiff structures. In this study, we developed a stretchable, high-density sEMG electrode array via layer-by-layer printing and lamination. The electrode offered a series of excellent human‒machine interface features, including conformal adhesion to the skin, high electron-to-ion conductivity (and thus lower contact impedance), prolonged environmental adaptability to resist water evaporation, and epidermal biocompatibility. This made the electrode more appropriate than commercial electrodes for long-term wearable, high-fidelity sEMG recording devices at complicated skin interfaces. Systematic in vivo studies were used to investigate its ability to classify swallowing activities, which was accomplished with high accuracy by decoding the sEMG signals from the chin via integration with an ear-mounted wearable system and machine learning algorithms. The results demonstrated the clinical feasibility of the system for noninvasive and comfortable recognition of swallowing motions for comfortable dysphagia rehabilitation.

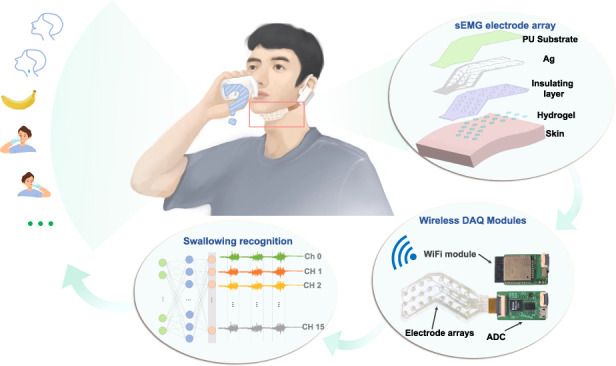

## Introduction

With continuous advancements in medical technology, the prevention and rehabilitation processes for diseases with long treatment cycles, such as strokes, are becoming major concerns^1,2^. The costs of hospital tests and treatments are frequently very high, making them uneconomical for protracted healing processes. An alternative strategy is to use portable monitoring devices to enable home disease monitoring. This model enables real-time monitoring of the patient’s health status, and the data are processed and saved via wireless transmission, and the results are presented with simple visualizations^3,4^. Since it is a noninvasive and easy-to-use technique, surface electromyography (sEMG) is often used for monitoring health and treating diseases. The measurement of the EMG signal depends mainly on the interface between the skin and the electronic acquisition circuit. However, the commercial Ag/AgCl electrodes used in existing sEMG acquisition devices are insufficient to establish a robust, conformal interface for long-term high-fidelity sEMG signal acquisition. The electrodes are typically made of nonstretchable materials and are electrically connected via metal buttons to ensure the stability of the device. When the skin is distorted by human activity, interface delamination occurs between the electrode and the skin because of the significant modulus difference. In addition, commercial electrodes add a gel serving as a binder and ionic medium, which leads to loss of signal quality due to dehydration over long periods of use^5,6^.

Fortunately, the rapid development of electronics based on flexible materials and structures offers new options for high-fidelity recording of bioelectrical signals. Flexible electron-based myoelectric electrodes have good compliance and biocompatibility and can build seamless and conformal skin-electrode interfaces, and some electrodes can also be stretched to a certain degree^7–12^. To eliminate motion artifacts arising from the different moduli for the skin and the electrode, stretchable electrodes provide soft conformal contact even during skin deformation. In general, there are two main design strategies for stretchable electrodes. One is based on an elaborate structure such as serpentine, where a nonstretchable conductor is spatially endowed with deformability^7,9,13–19^. The other category uses conductive fillers filled with intrinsically stretchable composites, such as hydrogels or Polydimethylsiloxane (PDMS) filled with silver nanosheets^11,20–23^. These methods are designed to improve the conformal contact of the flexible electrodes with skin exhibiting large curvature and complex textures. Curved skin interfaces frequently have complex muscle distributions. Examples include the larynx and the face, where there are many more small muscles than there are in the arm. The traditional bipolar disk sEMG acquisition mode can only acquire sEMG signals from one measured area, which may have multiple muscles or muscle groups superposed and does not accurately reflect the specific muscle to be analyzed. High-density surface electromyography (HD-sEMG) compensates for the shortcomings of conventional differential electrodes. It quantitatively analyzes the intensity and temporal sequence information from different muscles in space. Moreover, it enables accurate, comprehensive, and objective evaluation of the synergistic effects of different muscles during muscle activity. Kim et al. presented a reusable, multichannel sEMG sensor array that covered multiple muscles over relatively large areas. The stretchable structure and array design based on serpentine interconnections enabled it to acquire sEMG information from complex curved surfaces such as the back of the hand or the face^16^. Although dry electrodes can achieve stable, long-term monitoring, the lack of robust adhesion to the skin leads to inevitable relative slippage against the skin. Hydrogels can establish robust bioelectronic interfaces with properties close to those of human tissue as well as easy processability with which to engineer the mechanical strength, biological adhesion, or electrical conductivity^24–26^. For example, Gong et al. reported a neutral polyampholyte hydrogel (PA gel), which showed rapid, strong, and reversible adhesion to charged hydrogels or biological tissues through coulombic interactions with its self-adjustable surface^27^. For hydrogel-based on-skin electrodes, another important feature is the conductivity of the hydrogel. It can be enhanced by adding ions or by adding conductive fillers such as carbon nanotubes and conductive polymers^28–31^. In addition, to enable long-term monitoring of physiological signals, the resistance of the hydrogels to water loss can be enhanced by adding humectants in the network^32^. While it is a difficult task to accomplish mass production of the aforementioned dry electrodes or wet electrodes at an affordable price, they are meant to be used widely for physiological electrode detection as a replacement for commercial electrodes. This is due to the high cost and the need for sophisticated equipment for traditional MEMS-based manufacturing techniques. In this context, the continuous advancement in printing electronics with large-scale manufacturing methods, such as screen printing, offers a way to make highly adhesive flexible electrodes more widely available^33,34^.

**Fig. 1:**
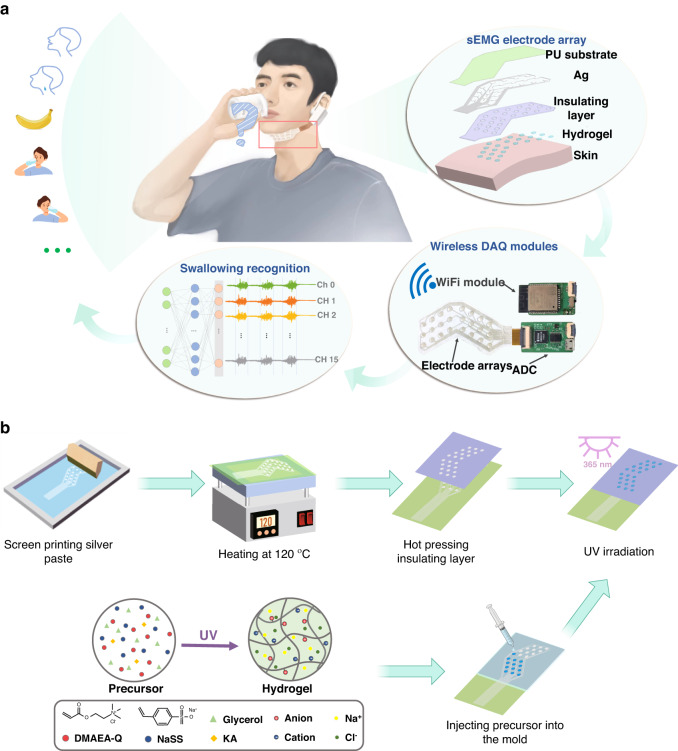
Schematic illustration of the HD-sEMG electrode patch. **a** HD-sEMG electrode patch enables swallowing classification. **b** A layer-by-layer printing and lamination fabrication method

In this work, we propose a stretchable and durable HD-sEMG electrode array patch to be worn on complicated skin interfaces, as shown in Fig. 1a. It enables long-term, stable monitoring of sEMG signals without interference caused by motion, even when the skin is substantially deformed. The electrode array uses stretchable silver electrodes as the signal transmission channels. They are first screening printed on a thin layer of commercially-available PU substrate and are then insulated with a second thin layer of double-adhesive wound dressing film. This sandwich structure ensures that the entire patch is stretchable and conformable. To improve ion-to-electron transduction, the electrode employs conductive glycerol-water polyampholyte hydrogels (GW-PA gels) at the electrode-to-skin interface. The GW-PA gels are deposited in situ and polymerized on silver electrodes to form GW-PA gel-based silver electrodes (GW-PA-Ag electrodes) capable of direct conductive contact between the patch and the skin. Moreover, since the GW-PA gel forms numerous electrostatic interactions and hydrogen bonds with biological soft tissues, the adherence of the patch to the skin is significantly improved. Altogether, these features allow the patch to be used for the high-fidelity recording of submandibular EMG signals. As a result, the GW-PA-Ag electrode exhibits a number of outstanding human‒machine interface qualities, such as 34.3 N m^–1^ adhesion to porcine skin, ultrastretchability to 960%, a skin-matched modulus of approximately 10 kPa, and a much lower skin interface impedance than commercial electrodes (due to the abundance of free ions in the gel). More importantly, this patch withstands a high strain of 80% without mechanical delamination between any of the layers or water loss during one week of EMG monitoring without deteriorating the signal quality (due to the introduction of glycerol in the gel network). To demonstrate its practical utility in the detection of EMG signals on subchinococcygeal and subglottic muscle groups, the patch was integrated with a 16 analog channel readout circuit and a WiFi module in an ear-mounted wearable device for the classification of swallowing activity. Using a deep learning algorithm, eleven different swallowing actions are recognized and categorized with 80% accuracy. This work holds promise for screening and treatment of dysphagia and offers a viable and affordable method for remote rehabilitation.

### Design of the swallowing recognition electrode array patch

Figure 1b shows the fabrication process featuring the layer-by-layer printing and laminating methods. The metal electrodes (5 mm in diameter) with conducting channels and readout interfaces were formed by first screen printing silver pastes on a thin layer of PU substrate and then annealing at 120 °C for 20 min. After that, a piece of off-the-shelf wound dressing film was mechanically patterned with a cutting plotter to create through-holes to expose the silver electrodes for hydrogel deposition. Medical dressing film was used as an adhesion and insulating layer as well as a supporting layer, as it is stretchable, biocompatible, and optically transparent. The two films were then hot pressed at 150 °C for 15 s to form strong mechanical bonds between them. Interestingly, the hot-pressed silver electrode exhibited improved electrical characteristics in terms of electrical resistance. The resistance of the silver electrode was approximately 27 ohms before it was hot pressed; however, after it was hot pressed, the resistance dropped to only 2 ohms, which was 13.5 times lower. Additionally, the resistance of the hot-pressed silver electrode was less than 100 ohms when stretched to 50% strain, while the resistance of the electrode before hot pressing exceeded 1000 ohms for the same stretch (Fig. S1, Supporting Information). The difference was attributed to the formation of a much denser microstructure in the silver electrode due to thermal lamination, which increased the electrical conductivity. Next, the GW-PA gels were deposited on silver electrodes by using a layer of patterned silicone mold, which enabled on-site, UV curing of the coating and formation of the desired human‒machine interface. The presence of glycerol and the electrostatic attraction of the GW-PA gel made its surface extremely sticky, which led to a robust interface between the gel and silver electrode. As shown in Fig. S2, Supporting Information, this interface withstood a large deformation generating 80% strain without interfacial delamination and presented a large interfacial toughness of 596 J/m^2^. Finally, the patch was trimmed into a v-shape that fit snugly against the submandibular to increase its wearability and obtain high-quality sEMG signals for swallowing activity recognition.

## Results and discussion

### Mechanical properties of the GW-PA gels

**Fig. 2:**
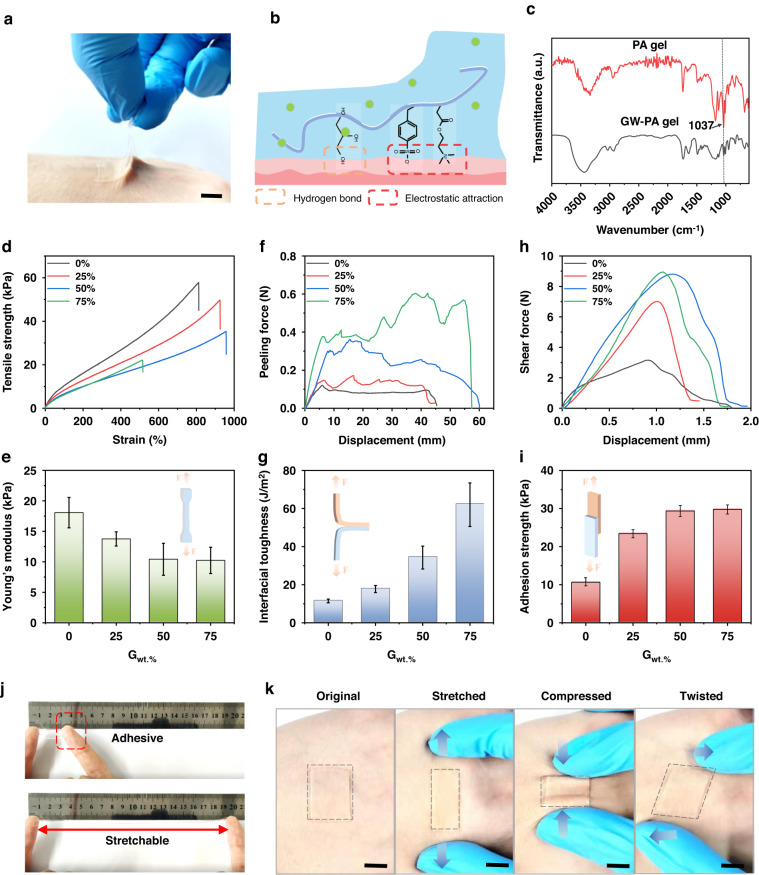
Mechanical properties of the GW-PA gels. **a** optical photograph showing tight bonding between the GW-PA gel and human skin; **b** adhesion mechanisms of GW-PA gels; **c** FTIR-ATR spectra of the hydrogel with glycerin (GW-PA) or without glycerin (PA); **d**, **e** tensile strength, **f**, **g** interfacial toughness, and **h**, **i** adhesion strength of the GW-PA gels as a function of the glycerin content (0%, 25%, 50%, 75%). **j**, **k** Visual demonstration of strong adherence between the GW-PA gel and human skin with considerable stretching, compression, and twisting, which did not show any sign of detaching from the skin. Scale bar: 10 mm

To eliminate any motion artifacts caused by unintentional slippage on the skin, epidermal electrodes usually require strong adhesion between the electrode and skin to ensure high-quality sEMG signals. In this work, this was achieved by using a GW-PA gel to form the adhesive, conductive electrode-to-skin interface. As shown in Fig. 2a, a thin layer of GW-PA gel, when peeled slowly, pulled up a layer of epidermis from the forearm instead of being delaminated from the forearm. In addition, it left no residue on the skin (Fig. S3, Supporting Information). A magnified view of the hydrogel attached to a layer of porcine skin showed that the gel seamlessly adhered to the complex texture of the porcine skin (Fig. S4, Supporting Information). The strong adhesion of the hydrogel to the skin tissue surface was due to the synergistic effect of electrostatic attraction and hydrogen bonding (Fig. 2b). The neutral GW-PA gel had equal numbers of positive and negative charges (positively charged ammonium and negatively charged sulfonate) randomly distributed, which provides nonspecific adhesion capacity^35^. Contact with positively or negatively charged objects causes charge redistribution on the gel surface. As a result, the charges in the gel flow to the opposing charged objects and create powerful electrostatic interactions^27^. The strong biological adhesion of the GW-PA gel was investigated with FTIR-ATR spectroscopy, and Fig. 2c shows the result. In contrast to the PA gel, which had a disappearing peak at 1037 cm^–1^, the GW-PA gel exhibited this peak, which was attributed to a C-O stretching vibration of glycerin, indicating the presence of glycerin in the gel network. Additionally, the high viscosity of glycerol promoted strong interfacial interactions, which also enhanced the intermolecular interactions^29^. More excitingly, due to nonspecific adhesion by the gel, it adhered to a variety of different materials (Fig. S5, Supporting Information). This explicitly showed that the GW-PA gel formed robust electrical interfaces with different electronic materials or biological tissues.

The mechanical properties of the GW-PA gels were optimized by adjusting the glycerol weight content. As shown in Fig. 2d, the tensile strengths of the GW-PA gels were inversely proportional to the glycerol content in the matrix. At 0% (w/w) glycerol content, the gel had a Young’s modulus of 18 kPa, which decreased to 13.8 kPa and 10 kPa for 25% and 50% (w/w) glycerol contents, respectively (Fig. 2e). The Young’s modulus stabilized at 10 kPa when the glycerol proportion was increased above 50% (w/w). The reduction in the Young’s modulus of the gel resulted from a reduction in the van der Waals forces among polymer molecules due to the plasticizing effect of the glycerol^36^. In contrast, the relationship between the stretchability of the gel and the glycerol ratio (over a range of 0–50% w/w) presented an inverse pattern, i.e., a larger glycerol proportion increased the stretchability of the gel. The maximum stretchability approached 960% at 50% (w/w) glycerol, although it decreased as more glycerol was added. Given that it has the lowest hardness of 10 kPa, which was similar to that of human soft tissue^37^, and the highest stretchability of 960%, the GW-PA gel with 50% (w/w) glycerol was chosen for construction of the GW-PA-Ag electrode.

The adhesion strengths of the GW-PA gels also depended on the glycerol weight content. They were determined via standard 180-degree peeling and lap-shear tests of interfacial toughness and adhesion strength, respectively. As depicted in Fig. 2f–j, the peeling force and shear force of the GW-PA gels and skin were proportional to the glycerol content. When the glycerol proportion was increased from 0% to 75% (w/w), the interfacial peeling force increased linearly from 11 J/m^2^ to 62 J/m^2^, while the adhesion strength of the shear force increased drastically from 10 kPa to 30 kPa. The increased adhesion seen when increasing the glycerol content was due to the dual effects of high glycerin viscosity and a lower Young’s modulus. The low Young’s modulus allowed the gel to stick to skin with a complicated texture, which increased the contact area and enhanced the adhesion strength. As a result, the gel exhibited strong adhesion and a low Young’s modulus, so it attached firmly to fingers and withstood uniaxial tension with up to 900% strain (Fig. 2j, Movie S1, Supporting Information). The adhesion dynamics of the GW-PA gel were qualitatively evaluated by placing the gel on the back of the hand or the metacarpophalangeal joint and observing skin adherence for a number of deforming and relaxing cycles. The former was performed by stretching, compressing, or twisting the peripheral skin surrounding the gel, while the latter was conducted by clenching and loosening the fist. As shown in Figs. 2k, S6 (Supporting Information), the GW-PA gel conformed to these shapes without mechanical disintegration or relative slippage.

### Electrical properties of the GW-PA-Ag electrodes

**Fig. 3:**
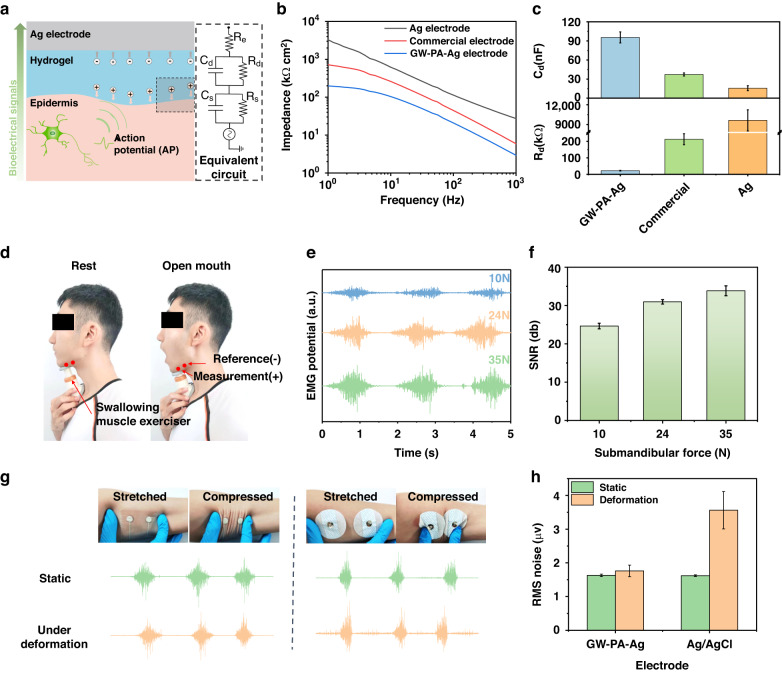
Electrical characterization with the GW-PA-Ag electrode. **a** Schematic representation of the sEMG signal traveling from the skin to the electrode with an equivalent circuit model; (**b**, **c**) Comparison of the contact impedances for three types of sEMG electrodes; (**d**) photograph of the GW-PA-Ag electrode used to measure sEMG signals from the submandibular of a participant practicing a swallowing muscle exercise; (**e**, **f**) results showing the amplitude and SNR of sEMG signals proportional to the submandibular force; (**g**, **h**) sEMG signal quality of the GW-PA-Ag electrode during cyclic deformation in comparison to the commercial electrode

As described previously, the GW-PA gel was polymerized and bonded in situ on the surface of the silver electrode to form the GW-PA-Ag electrode and served as the on-skin sEMG interface. Figure 3a shows a schematic illustration of a bioelectrical signal traveling between an electrode and the skin. In the equivalent circuit model, a pair of parallel capacitances Cs and resistances R_s_ represented the electrical characteristics of the stratum corneum and sweat glands of the skin, whereas a second pair of parallel capacitances C_d_ and R_d_ were those of the electrode-to-skin interface, in which C_d_ is the interfacial capacitance and R_d_ is the charge transfer resistance. In addition, R_e_ is the intrinsic resistance of the silver electrode. Given that C_s_ and R_s_ are constant and R_e_ is negligible, the total contact impedance of the equivalent circuit depended on that of the parallel C_d_ and R_d_. Meanwhile, the GW-PA gel that contained a large amount of electrolytes (e.g., Na^+^) acted as an ion conductive layer, which resulted in capacitive coupling of the ionic and electronic currents between the silver electrode and the skin^24^. As a result, the GW-PA gel generated a much larger interfacial capacitance C_d_ and a much lower impedance than a commercial Ag/AgCl electrode or a dry Ag electrode. Figure 3b shows the contact impedance plots (Bode plots) for each electrode set. All electrodes displayed a one-directional decrease in impedance over the frequency range 1 to 1000 Hz. This was consistent with the equivalent electrical model in Fig. 3a, which suggested that the interfacial capacitance played a major role in determining the electrode-skin impedance. More importantly, the GW-PA-Ag electrode exhibited the lowest electrode-skin impedance among all electrodes, whereas the Ag electrode displayed the highest impedance. The dry Ag electrode had a substantially larger contact impedance at a few MΩ than the wet electrodes because it lacked a wetting effect on the stratum corneum and electron-to-ionic conductivity. This significantly reduced its capacity for sEMG measurements. The electrochemical impedance spectra also confirmed this observation. As shown in Fig. S7 (Supporting Information), the Nyquist plot for the GW-PA-Ag electrode contact impedance contained a distinct semicircle with curved lines, while those of the commercial and Ag electrodes appeared as straight lines, implying much larger Nyquist semicircles. Fits of the Nyquist plots to the equivalent circuit model (Fig. 3a) gave a calculated interfacial capacitance of 95 nF and a calculated charge transfer resistance of 22 kΩ for the GW-PA-Ag electrode (Fig. 3c). Since the former was the largest and the latter was the smallest among all, their parallel value meant the GW-PA-Ag electrode had the smallest contact impedance and the highest sEMG quality.

sEMG signals from the swallowing muscles are often smaller than those from larger muscles, including those in the forearm. This poses a challenge for the acquisition of swallowing sEMG signals, as the sEMG signals may be drowned in noise when sEMG is used for activities with low muscle activity. We evaluated the performance of the GW-PA-Ag electrode by recording sEMG signals for different levels of submandibular force, since either mouth opening or swallowing requires the use of the targeted muscles in the submandibular region. As shown in Fig. 3d, this was accomplished by employing a pair of GW-PA-Ag electrodes as working and reference electrodes to measure the amplitudes and SNRs of the sEMG signals from a participant’s submandibular while he was performing a swallowing muscle exercise, which involved opening his mouth and compressing a spring completely under his jaw. Three springs were used, which produced forces of 10 N, 24 N, or 35 N when fully compressed. Figure 3e, f presents the sEMG signals generated by the submandibular muscles as a function of the muscle strength. At a low muscle strength, i.e., 10 N, the peak magnitude and SNR of the recorded sEMG signal were 150 µV and 25 dB, respectively. Then, the intensity of the sEMG signal increased approximately linearly as muscle strength was increased. For instance, at 24 N and 35 N, the peak magnitudes increased to 350 µV and 505 µV, respectively, which were 2.3 and 3.4 times those seen at 10 N. In addition, the SNRs increased to 30 and 34 dB, which were 1.2 and 1.36 times that seen at 10 N, respectively. This demonstrated that the GW-PA-Ag electrode was capable of detecting sEMG signals for different forces and detected a weak swallowing muscle force of 10 N with a high SNR (>24 dB).

A good sEMG electrode should recognize motion artifacts, as they are the main sources of interference when monitoring muscle activity signals on the body surface. Conventional sEMG electrodes encounter relative slippage between the skin and electrodes during human muscle action as a result of the stress mismatch. This disrupts the charge distribution at the interface and consequently results in motion artifacts^38^. For this reason, we evaluated the robustness of the GW-PA-Ag electrode by examining the signal quality for motion artifacts, which were manually created by cyclically stretching and compressing the skin around the electrode when performing a grip strength exercise of 50% MVC. As shown in Fig. 3g, the commercial electrodes moved away from the targeted position, but the GW-PA-Ag electrodes conformed tightly to the skin even during severe skin deformations. As a result, the baseline noise was significantly increased for the commercial electrodes. The root-mean squared (RMS) values of the baseline noise were quantitatively analyzed, and those of the GW-PA-Ag and commercial electrodes were compared. The results are shown in Fig. 3h. When the skin was at rest, the RMS value for the GW-PA-Ag electrode was 1.7 µV, which was slightly lower than that seen with the Ag/AgCl gel electrodes. The difference, however, grew larger when the skin was under tension or compressed. The RMS noise measured with the GW-PA-Ag electrode remained unchanged whether the skin was stretched or compressed. However, Ag/AgCl electrodes showed a substantially higher RMS value of 3.6 µV. These analyses indicated that the GW-PA-Ag electrodes tolerated motion artifacts at least twice those of the commercial electrodes.

### Long-term stabilities of GW-PA-Ag electrodes

**Fig. 4:**
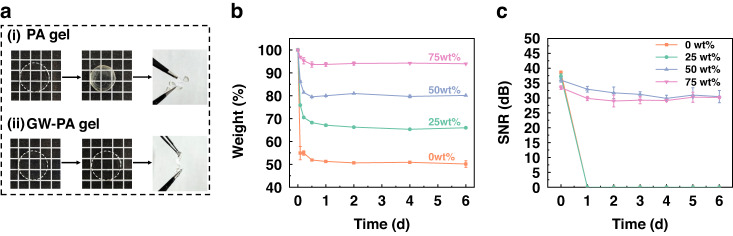
Long-term stability of the GW-PA-Ag electrode. **a** qualitative comparison of the mechanical strengths for the GW-PA and PA hydrogels after 7 days of drying; **b** time-dependent weight change of the GW-PA-Ag electrodes as a function of the glycerol content at a temperature of 25 °C and a humidity of 60%; **c** the PA-GW-Ag electrodes with 50% or 75% w/w glycerol maintained a SNR > 30 dB over 7 days

Due to the prolonged nature of rehabilitation treatments, an sEMG electrode with a long shelf life may provide patients with more practical and affordable therapy options. The abilities of GW-PA-Ag electrodes to retain weight and a high SNR over the long term were examined daily for a total of 7 days. As shown in Fig. 4a, the PA gel experienced a large decrease in water volume and significant volumetric shrinkage after 7 days of drying. This caused a problem in the structural integrity during low level bending. On the other hand, the GW-PA gel maintained considerable water (and showed negligible water loss) from network, so it tolerated a certain degree of mechanical deformation. The water retention capability of the GW-PA gel was attributed to the presence of glycerol, since glycerol is hygroscopic and forms hydrogen bonds with water to prevent evaporation. The effects of glycerol on the time-dependent drying kinetics of the GW-PA-Ag electrode were quantitatively evaluated as a function of the glycerol weight ratio. The electrode was kept in a programmable constant temperature and humidity chamber by fixing the temperature at 25 °C while varying the humidity from 40% to 80% with escalating steps of 20%^39,40^. As shown in Fig. 4b, the electrode embedded with a high volume of glycerol (i.e., 75% w/w) lost 7% of its own weight after 1 day but maintained this level for the remaining days, whereas the losses increased to 20%, 32%, and 50% when the glycerol ratio was decreased to 50%, 25%, and 0% w/w, respectively. Since oven drying of the PA gel gave a completely dried mass fraction of approximately 45%, the sample without glycerol basically lost all of its water. Moreover, the SNR values of the GW-PA-Ag electrodes showed similar time-dependent trends. This was clear when the SNRs dropped sharply after one day of drying and remained stable for the following days (Fig. 4c**)**. Among the three electrodes, the electrode with 50% glycerol had the highest SNR of 28 ± 1.1 dB lasting for 7 days, while the electrode with 0% glycerol had the highest SNR of 38 ± 0.5 dB on Day 0. This was because the latter had the most water in its network, which enabled faster ion migration and, consequently, gave the strongest sEMG signal. However, since it was nearly totally dried after one day and was unable to collect the sEMG signals, the scenario did not endure for more than one day. As a result, the sEMG SNR dropped significantly to 0 dB. This also occurred for the electrode with 25% glycerol. In addition, in decreasing or increasing the humidity to 40% or 80%, respectively, similar drying patterns were observed for all of the electrodes with variations of no more than 5% (Fig. S8, Supporting Information). These findings suggested that the electrode with a glycerol ratio greater than 50% functioned in a wide range of settings.

### Biocompatibility of the GW-PA-Ag electrodes

To ensure the safety of the materials used in the GW-PA-Ag electrode and practical use for wearable applications, the biocompatibilities of the GW-PA hydrogel and GW-PA-Ag electrode were evaluated with human immortalized keratinocytes (HaCaTs) cells that were in close contact with the on-skin electrodes. For this test, human HaCaTs were cultured directly on the materials for 24 h or 72 h. The cell survival rates are shown in Fig. S9, Supporting Information. At 24 h, more than ∼95% of the cells were viable on both the GW-PA hydrogel and the GW-PA-Ag electrode. After 72 h of direct exposure, the percentages of viable cells were maintained at the same level, and there was no statistically significant difference in the percentages of viable cells regardless of the substrate materials (GW-PA hydrogel or GW-PA-Ag electrode) when compared to that of the control. Overall, the cell culture results indicated that the GW-PA-Ag electrode was highly biocompatible with human skin cells (with a notable lack of acute toxicity), confirming the suitability of the stretchable GW-PA-Ag electrode for a broad range of on-skin applications. Moreover, sandwiching of the silver electrode between the hydrogel and PU substrate meant that this structure guaranteed little or no leakage of the silver electrode and little or no cytotoxicity to the surrounding tissues, which in turn enhanced the biocompatibility of the assembled electrode. It should be noted that only the hydrogel and electrode-and not the silver electrode-were tested.

### GW-PA-Ag electrode patch for swallowing detection

**Fig. 5:**
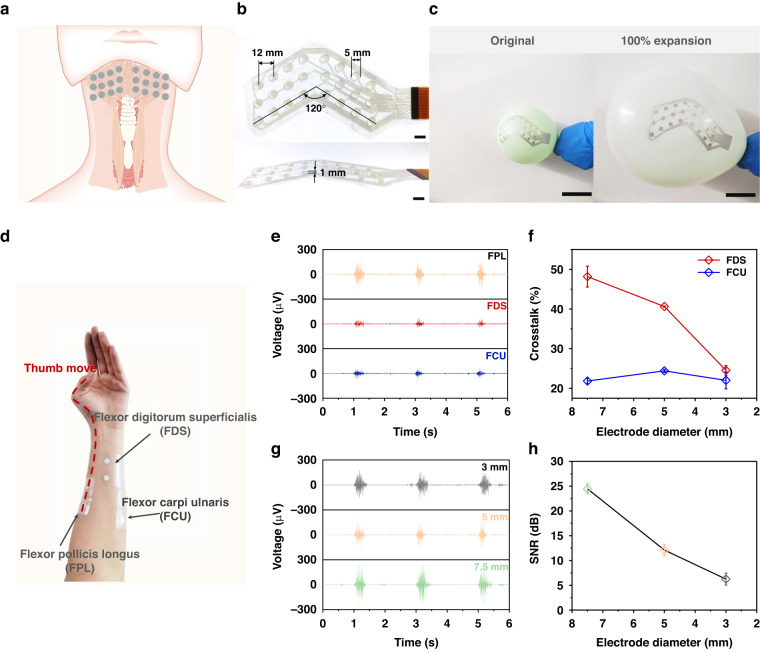
GW-PA-Ag electrode patch for swallowing detection. **a** schematic showing the patch placed on the swallowing muscles; **b** photographs of the patch connected with a 16-channel readout circuit. Scale bar: 5 mm. **c** Qualitative characterization of the patch under tension with an inflated balloon. Scale bar: 50 mm. **d** Photographs showing the crosstalk of the patch; **e** sEMG signal magnitudes for the FPL, FDS, and FCU muscles when the thumb moved, **f** and the crosstalk rate of the latter two on the FPL muscle as a function of electrode diameter. The **g** magnitude and **h** SNR of sEMG signals measured on the FPL as a function of electrode diameter

According to previous studies on the correlation between sEMG activities and swallowing^41^, the subchinococcygeal and subglottic muscle groups are the active muscles that generate swallowing action, and their sEMG signals can, to some extent, reflect the ease of hyoid elevation movements^42^. To precisely cover these areas for submandibular sEMG measurements, a single electrode was engineered into a patch with a patterned array of 24 electrodes. Figure 5a is a schematic view of a V-shaped patch applied to the subchin muscle groups associated with swallowing activities. The detailed layout of the patch is shown in Fig. 5b. Sixteen of the 24 circular contacts (5 mm diameters and 12 mm center-to-center gaps) were employed as working electrodes, and the rest were reference electrodes. To prevent motion artifacts from impairing the sEMG signal, the array patch must adhere tightly to the target skin even while it experiences significant skin deformation brought by swallowing. Prior to the practical demonstration, we attached the patch to a balloon and examined deformation during inflation to evaluate the adherence capacity. As shown in Fig. 5c (also seen in Supplementary Movie 2), no delamination or relative slippage was observed between the patch and balloon when the balloon was inflated to a large volume with a 100% increase in diameter. This demonstrated the conjugated adhesive strength of the GW-PA-Ag electrode and the insulating adhesion layer. It also revealed the excellent stretchability of the patch, which allowed it to withstand 100% strain without mechanical disintegration. In summary, this patch exhibited exceptional mechanical properties for adherence, flexibility, and stretchability.

The electrode size is an important parameter allowing an HD-sEMG patch to obtain accurate, high-quality sEMG signals. This was evaluated by placing a pair of GW-PA-Ag electrodes on the participant’s forearm and testing their signal quality as a function of electrode size (i.e., diameter), as shown in Fig. 5d. The signal quality was assessed with the signal magnitude and crosstalk rate, the latter of which was determined by the ratio between the signal amplitudes recorded for the inactivated and activated muscles^43^. The crosstalk rate represents the level of contamination of the tested muscle’s sEMG signal by adjacent muscles. In the test, two electrodes were fixed upon the flexor digitorum longus (FPL) muscle, while another two pairs of electrodes were placed on top of the flexor digitorum longus (FDS) and flexor digitorum ulnaris (FCU) muscles. By comparing the sEMG amplitude for the FDS or FCU to that for the FPL, it was possible to determine the crosstalk rate because only the FPL was activated when the thumb moved. Figure 5e and S10 of the Supporting Information show the strengths of the sEMG signals recorded from the three muscle groups when the thumb was bent. As predicted, the FPL had the largest sEMG amplitude with electrode diameters between 3 and 7.5 mm, while the FDS and FCU had significantly lower amplitudes. Figure 5f presents the crosstalk rate of the FDS or FCU with the FPL muscle. When using a 7.5 mm diameter electrode, the crosstalk rate for the FDS and the FPL was 48%; this dropped to 41% and 25%, respectively, when the electrode diameter was reduced to 5 mm and 3 mm. The crosstalk rate from the FCU to the FPL remained at a low level of approximately 22% regardless of the electrode diameter, since the distance between the FCU and FPL was greater than that between the FDS and FPL. The electrode size also affected the signal quality from the targeted muscle. As shown in Fig. 5g, h, both the sEMG signal amplitude and the SNR for the FPL increased as the electrode diameter was increased. The former had a peak value of 165 µV, and the latter had an SNR of 25 dB when utilizing a 7.5 mm diameter electrode; both were the highest of those determined. Given that the electrode with a 5 mm diameter had the most balanced performance, i.e., a crosstalk rate 41% lower than the 48% rate seen with a 7.5 mm diameter and an SNR 12 dB higher than the 6.2 dB SNR seen with a 3 mm diameter, it was selected for the remaining experiments in this study.

### Characterization of the swallowing actions

**Fig. 6:**
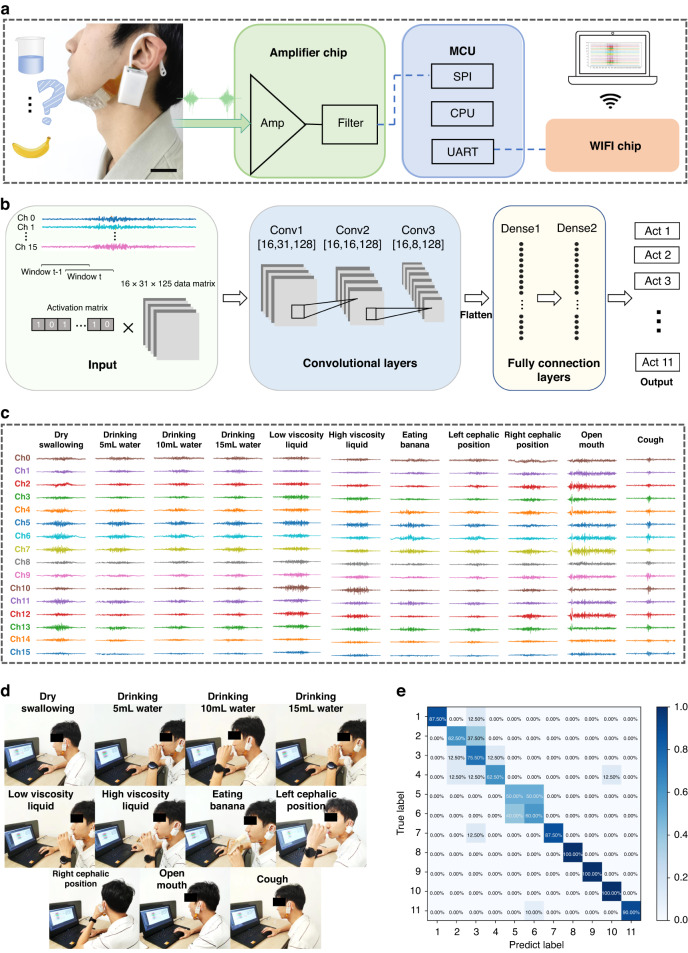
Swallowing activity classification. Schematic illustration of (**a**) the swallowing recognition system with (**b**) the machine learning algorithm. **c** Raw sEMG signals for (**d**) 11 swallowing actions, each with 16-channel inputs. **e** Confusion matrix of the swallowing classification for the 11 swallowing actions

To classify the swallowing actions, a wearable, wireless sEMG acquisition system was developed by connecting the patch to a 16 analog channel readout circuit with a customized (flexible printed circuit) FPC cable (Fig. 6a). The readout circuit used an RHA2116 chip (Inten Technologies) to perform signal amplification and analog-to-digital conversion. Once the system captured the sEMG signal, it transmitted the data to a convolutional neural network (CNN)-based framework on a host computer. As shown in Fig. 6b, an algorithm was developed to recognize swallowing activities from the 16-channel sEMG data (each 2 s long). Before the training, the raw data required preprocessing, mainly filtering. This was achieved by applying a 20–500 Hz bandpass filter, a set of 50 Hz power frequency notch filters and multipliers, and wavelet noise reduction in series (Fig. S12a, Supporting Information). As seen in Fig. S12b-c, before filtering, the sEMG signal showed a significant amount of baseline noise and motion artifacts within a frequency range of 500 Hz as well as over 60 μV^2^/Hz of industrial frequency interference at 50 Hz. However, these interferences were successfully removed by denoising (Figure S12d-e, Supporting Information). After noise reduction, the data were segmented with a 125 ms window providing a 62.5 ms overlap between each segment. This generated 31 segments, each with 125 sampling points. If the averaged amplitude of a segment was greater than 30% of the maximum among the 31 segments, it was considered to be active. Eventually, these processes created input with a size of 16 × 31 × 125 for the CNN framework. The CNN model consisted of three convolutional layers, two pooling layers, and two fully connected layers. The convolutional layers with residual blocks extracted features and reused them to enhance the network performance, and the pooling layers downsampled the data to remove the redundant information. After that, the fully connected layers with 512 nodes classified the features via the regression method and mapped the features into vectors via nonlinear transformations^44^. To classify the eleven swallowing actions, the softmax layer was employed to map the 512-dimensional vectors to a length of 11 vector values. Note that Hd-sEMG data from different subjects were scrambled for model training, which was designed to improve the ability to generalize unseen data.

Characterization of the swallowing motion included eleven swallowing motions, including dry swallowing, drinking 5 ml of water, drinking 10 ml of water, drinking 15 ml of water, a low viscosity liquid, a high viscosity liquid, eating a banana, the left cephalic position, the right cephalic position, an open mouth, and a cough. Among the eleven, dry swallowing and drinking 5/10/15 ml of water were used to represent swallowing different volumes of foods, while the low/high viscosity liquids (5 ml) and bananas (5 g) were used to represent swallowing various types of foods. The viscous liquids were obtained by varying the proportion of edible cellulose dissolved in water. In this context, the first seven swallowing actions involved the physical difficulties of ingesting foods with various volumes or formations, which are usually employed as indicators of the severity of dysphagia. The remaining four of the eleven were chosen to simulate abnormal swallowing conditions, in which the left cephalic or right cephalic position was achieved by turning the head to the side at a 45° angle and swallowing 5 ml of water. Figure 6c, d presents the raw EMG signals recorded in real time^45^. The EMG signals for the open mouth and cough were distinctly different from those for normal swallowing movements in terms of the amplitudes and durations. Meanwhile, the sEMG energy maps for swallowing with the three head positions revealed that when swallowing normally, the muscle activities on both sides of the lower jaw were nearly symmetrical, whereas when the head was turned to one side, the muscle activities on this side were inhibited (Fig. S13, Supporting Information).

Five volunteers aged 22 to 27 and without recorded medical diseases were asked to perform the eleven swallowing actions for 2 s and repeat them 10 times. To position the electrode, the participant was required to sit comfortably to keep the neck relaxed and avoid interference from other muscle activities. Then, the electrode was placed on the lower jaw so that its edge coincided with the mandibular medial edge. Note that the target skin was cleaned carefully with alcohol before each measurement. A total of 550 groups of data were gathered, and 60% of them were used to train the model, 20% to validate it, and the remaining 20% to test it. Figure 6e shows that our model achieved a high average classification accuracy of 80%. Among the eleven actions, the first seven, i.e., for foods with different volumes or formations, had low classification accuracies, among which (1) the two viscous foods were the most difficult to identify and gave the lowest classification accuracy of approximately 55%, and (2) the banana had the highest identification accuracy of 87.5%. Given that both the low- and high-viscosity foods come in the form of 5 ml liquids, it was possible to draw the following conclusions: (1) our system worked well in the identification of foods with different volumes or forms (liquid versus solid); and (2) in contrast to swallowing different food volumes, the different food types with varying viscosities shared more similar swallowing patterns based on the sEMG amplitude or duration, particularly for liquid foods. This made it difficult to distinguish between them. More importantly, the system successfully identified the four abnormal swallowing movements with accuracies higher than 87%, demonstrating its potential for use in the classification of dysphagia.

## Conclusion

In summary, this work developed a stretchable, durable HD-sEMG patch that was used for the classification of swallowing activities on complex epidermal surfaces. The patch was prepared with scalable, layer-by-layer printing and lamination to fabricate an array of GW-PA-Ag electrodes sandwiched between two thin PU films, which was used as a human‒machine interface to measure the sEMG data from targeted muscles. The fabricated electrode exhibited an excellent electrophysiological interface with an adhesion strength of 22.8 N m^−1^, an ultrastretchability of 1000%, a skin-matched modulus of approximately 10 kPa, and long-term stability of the SNR measuring no less than 30 dB over one week in an open environment. Compared to a commercial Ag/AgCl electrode, this electrode had a much lower contact impedance in the sEMG frequency range of 1 to 1000 Hz and half the noise baseline with large skin deformations. In the practical demonstration, this patch was applied with a trained CNN model for the recognition of eleven swallowing activities, three of which involved actual digestion of food, while four imitated abnormal swallowing motions. A high average classification accuracy of 80% was achieved, indicating the potential of this system for use in diagnosing dysphagia.

### Experimental section

#### Materials

Sodium *p*-styrenesulfonate (NaSS, 90 wt%) was purchased from Sigma‒Aldrich, Shanghai, China. Dimethylaminoethylacrylate quaternized ammonium (DMAEA-Q, 80 wt%) and 3-(methacryloylamino)propyltrimethylammonium chloride (MPTC, 50 wt%) were purchased from J&K Chemical Ltd. Glycerol (≥99%) and *α*-ketoglutaric acid (*α*-keto) were purchased from Sinopharm Chemical Reagent Co. Ltd. Stretchable conductive silver pastes (EM-1046) were purchased from Guang Zhou City Silver Well Trading Co., Ltd.‍ All reagents were of analytical grade and were used as received. Deionized water (DI, 18.3 MΩ) was used in all experiments.

### Preparation of the GW-PA gels

The P(NaSS-*co*-DMAEA-Q) hydrogel was prepared via free radical polymerization. First, the anionic monomer (NaSS), cationic monomer (DMAEA-Q), and photoinitiator (*α*-keto) were mixed with the water-glycerol binary solvent to form a pregel solution. The total ionic monomer concentration (CM) and the molar fraction of the anionic monomer were fixed at 2.3 M and 0.487, respectively, and the mole fractions of the initiators were 0.10 mol% relative to the CM. Different GW-PA gels were prepared by changing the mass fraction of glycerol in the binary solvent. The gels used for testing were prepared as follows: the pregel solution was injected into a reaction cell consisting of a pair of glass plate walls and a silicone spacer (10 cm × 10 cm × 1 mm). The gel was then polymerized for 5 h with an ultraviolet lamp (365 nm, 4 W cm^−2^) at room temperature. The hydrogels for sEMG measurements were polymerized in situ on the surface of the silver electrode with a layered array of patterned silicone film, and the rest of the process was the same as above.

### Electromechanical characterization

The interfacial toughness of the GW-PA gel was measured against porcine skin with the standard 180-degree peel protocol (ASTM F2256). It was determined by multiplying the averaged platform forces at the stable peel state and dividing it by the adhesion width. To measure the shear strength, the adhered samples were tested with the standard lap-shear test (ASTMF2255). The shear strength was determined by dividing the maximum force by the adhesion area. All samples were prepared with dimensions of 50 mm × 15 mm × 1 mm (length × width × thickness). The hydrogels were bonded with poly(ethylene terephthalate) (PET) films using a cyanoacrylate adhesive to prevent the gel from being stretched during adhesion testing. Before the tests, a 5 N preload was placed on the sample for 60 s. All tests were performed at ambient temperature with a universal testing machine (HZ-1004A, China) with a constant tensile speed of 50 mm/min^–1^. Uniaxial testing of the hydrogel was performed with the standard tensile test (ASTMF2258) to measure the tensile strength. Before the tests, the samples were cut into dumbbell shapes (gauge length *l* (12 mm) × width *w* (2 mm) × thickness *t* (1 mm)). The tests were performed at ambient temperature with a stretching velocity of 100 mm min^−1^. The Young’s modulus, *E*, was calculated from the initial slope of the stress−strain curve at a strain within 10%. Each type of experiment was performed in triplicate.

The electrode-skin impedance was measured with an electrochemical analyzer (CHI627D, Shanghai Chenhua, China) with a three-electrode system. During the measurement, the reference and counter electrodes were placed at distances of 3 cm and 6 cm from the working electrode, i.e., the fabricated GW-PA-Ag electrode. To cover the range of the sEMG signals, the scanning frequency range was set from 1 to 1000 Hz. For the long-term stability tests, the hydrogel electrodes were stored in a programmable constant temperature and humidity chamber with a temperature of 25 °C and a humidity of 60% before the experiments were performed. The single-electrode sEMG data acquisition experiment was performed with a commercial Noraxon wireless sEMG acquisition system. The signal-to-noise ratio (SNR) was calculated as follows:

1 $${SNR}({db})=20\times {\log }_{10}\frac{\sqrt{{\sum }_{k=1}^{N}{V}_{{singal}(k)}^{2}}}{\sqrt{{\sum }_{k=1}^{N}{V}_{{noise}(k)}^{2}}}$$

### Biocompatibility characterization

The GW-PA and GW-PA-Ag electrodes were cocultured with human immortalized keratinocyte (HACAT) cells to evaluate their cytotoxicities. Pristine Dulbecco’s modified Eagle’s medium (DMEM) served as the negative control. After sterilization via UV irradiation, the samples were added to the medium at a ratio of 10 mg/ml and extracted for 24 h. HACATs were seeded in 96-well plates at a density of 8 × 10^3^ cells/well, and the DMEM was supplemented with 10 vol % fetal bovine serum (FBS). A CCK-8 assay was performed to investigate the 24- or 72-h viability of the cells quantitatively. In addition, the absorbance was measured at 450 nm with a microplate reader (Invigentech, IV08-100).

### Ethical declaration

Ethics approval and informed consent were obtained from all participants, and the protocol was approved by the Maoming People’s Hospital Department of Ethics Committee (PJ2020MI-K179-01). The study was carried out under the Helsinki Declaration.

### Supplementary information

supporting information

supporting video S1

supporting video S2
