# Correction: Stretchable and durable HD-sEMG electrodes for accurate recognition of swallowing activities on complex epidermal surfaces

**DOI:** 10.1038/s41378-023-00621-0

**Published:** 2023-11-24

**Authors:** Ding Zhang, Zhitao Chen, Longya Xiao, Beichen Zhu, RuoXuan Wu, ChengJian Ou, Yi Ma, Longhan Xie, Hongjie Jiang

**Affiliations:** 1 Shien-Ming Wu School of Intelligent Engineering, South China University of Technology, Guangzhou, 511442 P. R. China; 2 School of Biomedical Sciences and Engineering, South China University of Technology, Guangzhou, 511442 P. R. China

**Keywords:** Electrical and electronic engineering, Materials science

Correction to: *Microsystems & Nanoengineering*

10.1038/s41378-023-00591-3 published online 18 September 2023

After the publication of this article^1^, it was brought to our attention that two references need to be added in the article, the necessary changes are as follows:

**Correction 1:**

The below description of “In order to collect sEMG signals from the suprahyoid and infrahyoid muscles, Makoto et al. used a flexible PCB (printed circuit board) multichannel sEMG array. Based on temporal and spatial data, they examined the muscular synergism during swallowing motions and presented a machine learning method to categorize swallowing patterns^24,25^.” will be added between the sentences of “Moreover, it enables accurate, comprehensive, and objective evaluation of the synergistic effects of different muscles during muscle activity.” and “Kim et al. presented a reusable, multichannel sEMG sensor array that covered multiple muscles over relatively large areas.” on page 2.

**Correction 2:**

- A published work by “ Suzuki, M. et al. Swallowing pattern classification method using multichannel surface EMG signals of suprahyoid and infrahyoid muscles. *Adv. Biomed. Eng*. **9**, 10–20 (2020).” was included as the new citation No. 24.
- The original reference No. 42 of “Murakami, C., Sasaki, M., Shimoda, S. & Tamada, Y. Quantification of the swallowing mechanism through muscle synergy analysis. *Dysphagia* **38**, 1–17 (2022).” became the updated reference No. 25.
- Overall, the citations from No. 24 to No. 46 were updated as follows:

24. Suzuki, M. et al. Swallowing pattern classification method using multichannel surface EMG signals of suprahyoid and infrahyoid muscles. *Adv. Biomed. Eng*. **9**, 10–20 (2020).

25. Murakami, C., Sasaki, M., Shimoda, S. & Tamada, Y. Quantification of the swallowing mechanism through muscle synergy analysis. *Dysphagia* **38**, 1–17 (2022).

26. Yang, G. et al. Adhesive and hydrophobic bilayer hydrogel enabled on-skin biosensors for high-fidelity classification of human emotion. *Adv. Funct. Mater*. **32**, 2200457 (2022).

27. Han, Q. et al. Hydrogel nanoarchitectonics of a flexible and self-adhesive electrode for long-term wireless electroencephalogram recording and high-accuracy sustained attention evaluation. *Adv. Mater*. **35**, 2209606 (2023).

28. Pan, L. et al. A compliant ionic adhesive electrode with ultralow bioelectronic impedance. *Adv. Mater*. **32**, e2003723 (2020).

29. Roy, C. K. et al. Self-adjustable adhesion of polyampholyte hydrogels. *Adv. Mater*. **27**, 7344–7348 (2015).

30. Yuk, H. et al. 3D printing of conducting polymers. *Nat. Commun*. **11**, 1604 (2020).

31. Han, L. et al. Mussel-inspired adhesive and conductive hydrogel with long-lasting moisture and extreme temperature tolerance. *Adv. Funct. Mater*. **28**, 1704195 (2018).

32. Tang, H. et al. In situ forming epidermal bioelectronics for daily monitoring and comprehensive exercise. *ACS Nano* **16**, 17931–17947 (2022).

33. Cheng, S. et al. Ultrathin hydrogel films toward breathable skin-integrated electronics. *Adv. Mater*. **35**, e2206793 (2023).

34. Hsieh, J. C. et al. A highly stable electrode with low electrode-skin impedance for wearable brain-computer interface. *Biosens. Bioelectron*. **218**, 114756 (2022).

35. Zavanelli, N. & Yeo, W. H. Advances in screen printing of conductive nanomaterials for stretchable electronics. *ACS Omega* **6**, 9344–9351 (2021).

36. Jain, V., Ochoa, M., Jiang, H., Rahimi, R. & Ziaie, B. A mass-customizable dermal patch with discrete colorimetric indicators for personalized sweat rate quantification. *Microsyst. Nanoeng*. **5**, 29 (2019).

37. Huang, Y. et al. Strong tough polyampholyte hydrogels via the synergistic effect of ionic and metal–ligand bonds. *Adv. Funct. Mater*. **31**, 2103917 (2021).

38. Wu, L., Li, L., Qu, M., Wang, H. & Bin, Y. Mussel-inspired self-adhesive, antidrying, and antifreezing poly(acrylic acid)/bentonite/polydopamine hybrid glycerol-hydrogel and the sensing application. *ACS Appl. Polym. Mater*. **2**, 3094–3106 (2020).

39. Yuk, H., Wu, J. & Zhao, X. Hydrogel interfaces for merging humans and machines. *Nat. Rev. Mater*. **7**, 935–952 (2022).

40. Heikenfeld, J. et al. Wearable sensors: modalities, challenges, and prospects. *Lab Chip* **18**, 217–248 (2018).

41. He, S., Cheng, Q., Liu, Y., Rong, Q. & Liu, M. Intrinsically anti-freezing and anti-dehydration hydrogel for multifunctional wearable sensors. *Sci. China Mater*. **65**, 1980–1986 (2022).

42. Edition, A. C. I. et al. Polyzwitterionic hydrogels for efficient atmospheric water harvesting. *Angew. Chem. Int. Ed*. **61**, e202200271 (2022).

43. Lee, Y. et al. Soft electronics enabled ergonomic human-computer interaction for swallowing training. *Sci. Rep*. **7**, 46697 (2017).

44. Farina, D., Merletti, R., Indino, B., Nazzaro, M. & Pozzo, M. Surface EMG crosstalk between knee extensor muscles: experimental and model results. *Muscle Nerve* **26**, 681–695 (2002).

45. Srinivasu, P. N. et al. Classification of skin disease using deep learning neural networks with MobileNet V2 and LSTM. *Sensors* **21**, 2852 (2021).

46. Zhu, M. et al. Evaluation of normal swallowing functions by using dynamic high-density surface electromyography maps. *Biomed. Eng. Online* **16**, 133 (2017).
