# Supplementary material for: Stretchable and durable HD-sEMG electrodes for accurate recognition of swallowing activities on complex epidermal surfaces
Source: Microsyst Nanoeng. 2023 Sep 18;9:115. doi: 10.1038/s41378-023-00591-3 (PMC10507084; doi:10.1038/s41378-023-00591-3)
Supplement: Supplementary file 1 — supporting information [file 41378_2023_591_MOESM1_ESM.docx]

**Supporting Information**

Stretchable and Durable HD-sEMG Electrodes for Accurate Swallowing Activity Identification and Classification on Complex Epidermal Surfaces

Ding Zhang^a^, Zhitao Chen^a^, Longya Xiao^a^, Beichen Zhu^a^, RuoXuan Wu^b^, ChengJian Ou^a^, Yi Ma^a^, Longhan Xie*^a^, Hongjie Jiang*^a^


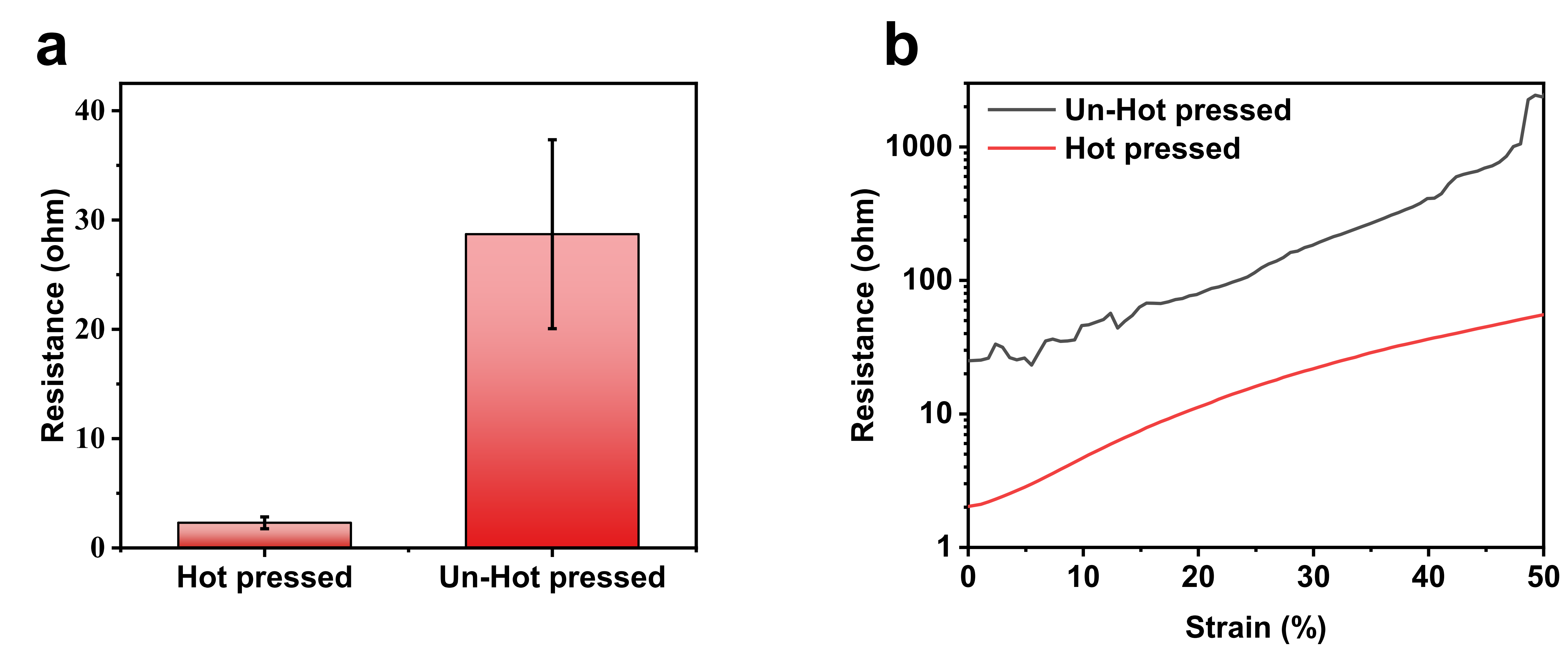


**Figure S1**. Electrical characteristics of the thermally compressed silver electrode in comparison to the uncompressed one.


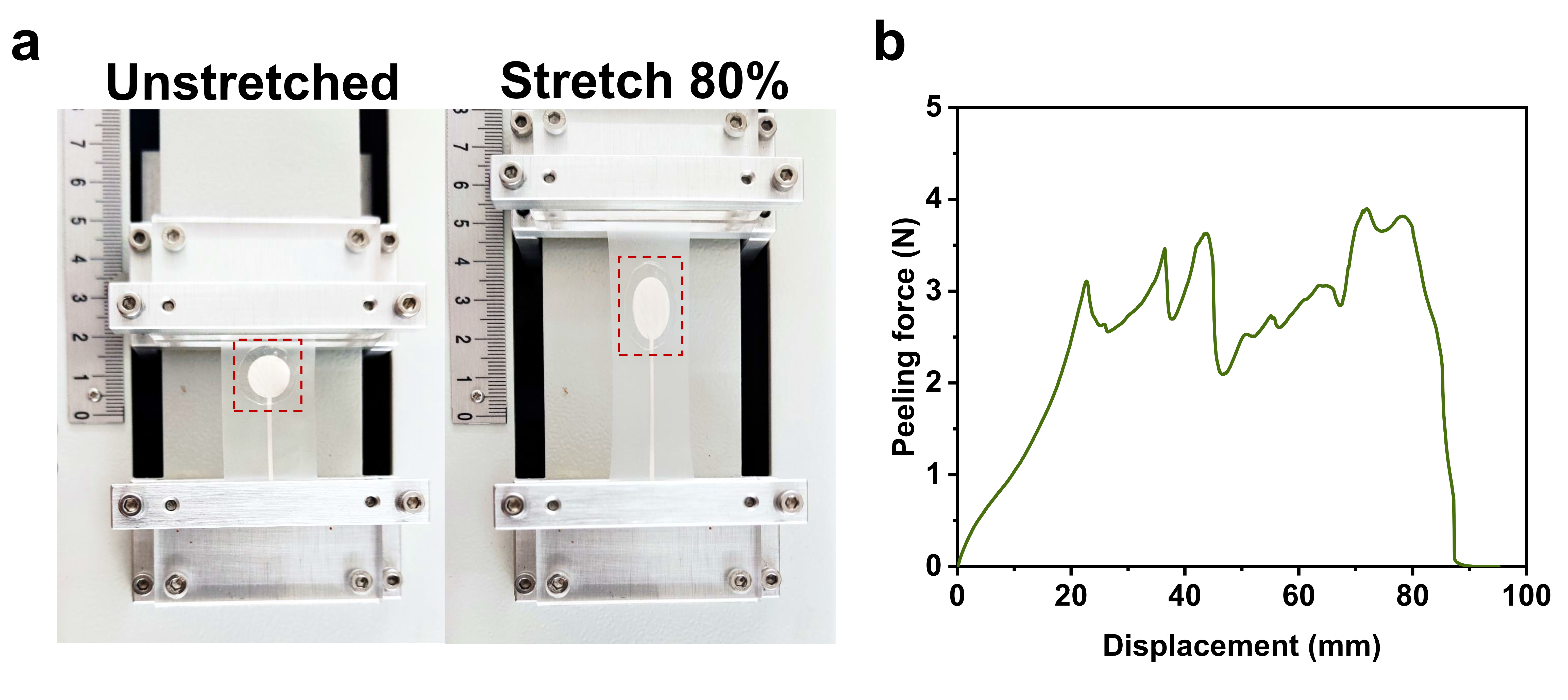


**Figure S2**. a) The bonding characterization of the GW-PA gel to the silver electrode showing a strong interfacial bonding between them able to withstand 80% deformation with a strong adherence strength of 596 J/m^2^.

**
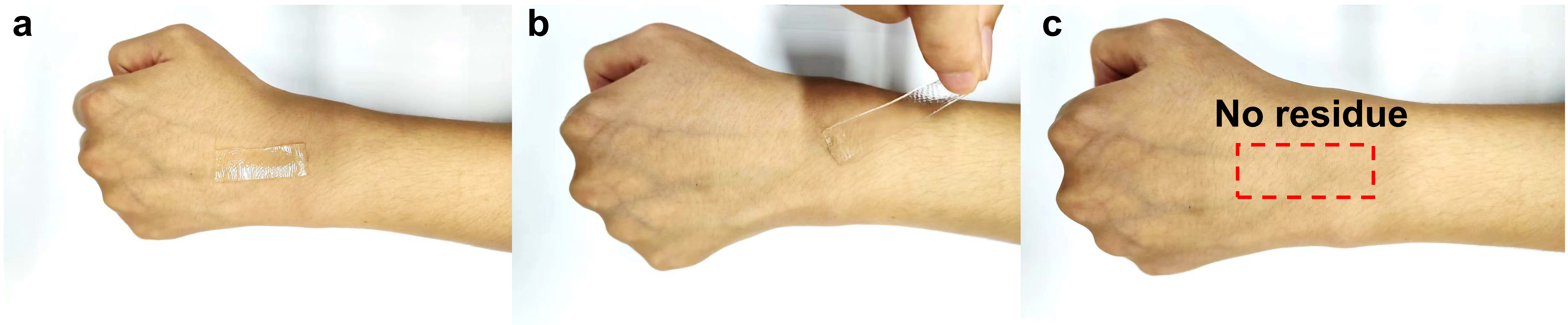
**

**Figure S3**. GW-PA gel can be removed from the skin without leaving any residue.

**

**

**Figure S4**. Microscopic optical images of hydrogel and pig skin conformal adhesion.

**

**

**Figure S5**. Adhesion ability of GW-PA gel to different materials.


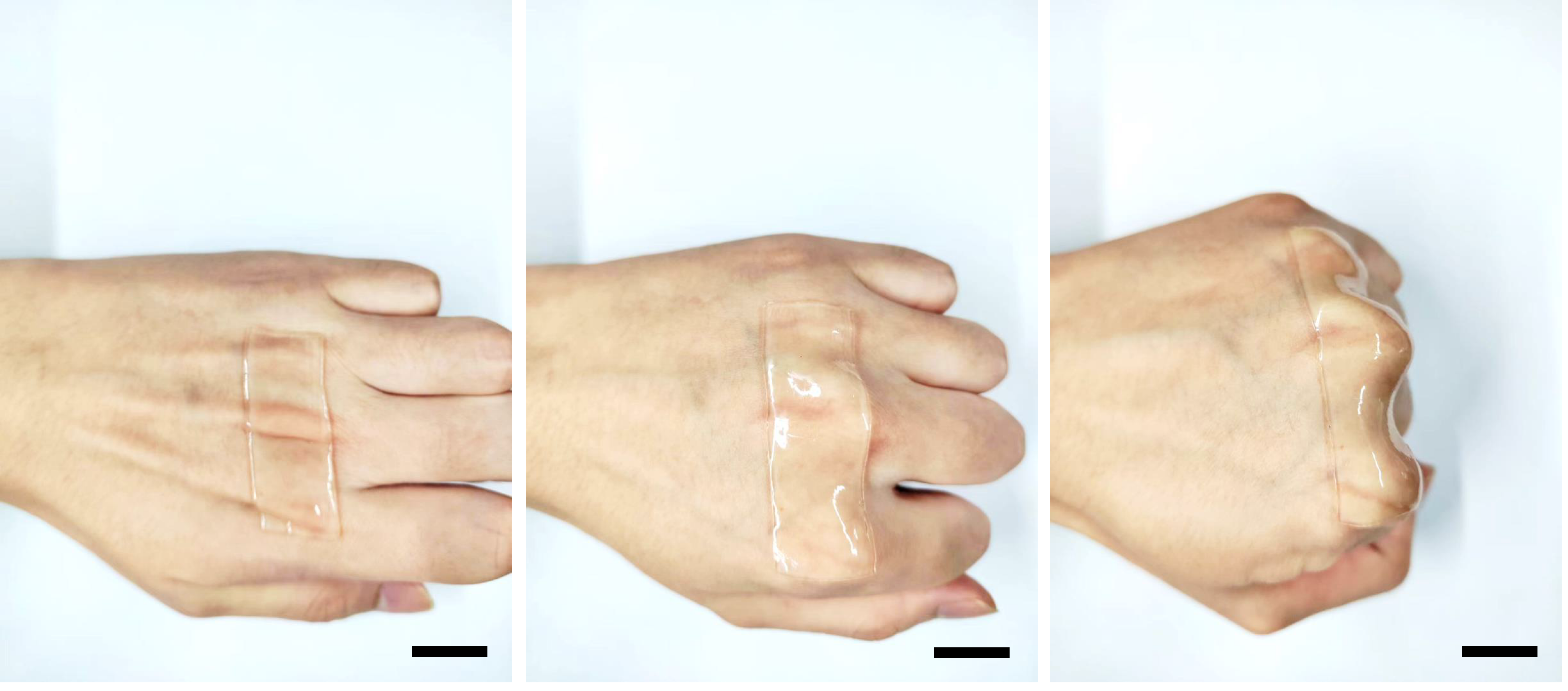


**Figure S6**. Adhesion ability of GW-PA gel to different hand activities.


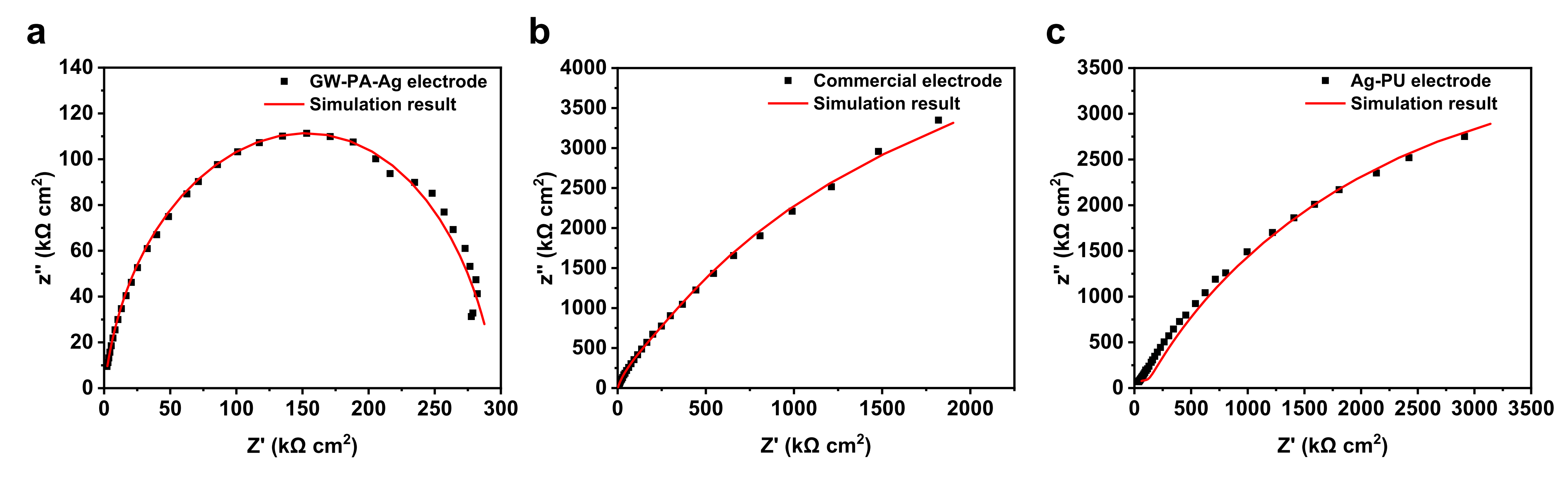


**Figure S7**. Nyquist plots measured by a) GW-PA-Ag electrode, b) Commercial electrode, c) as well as the corresponding simulation results.


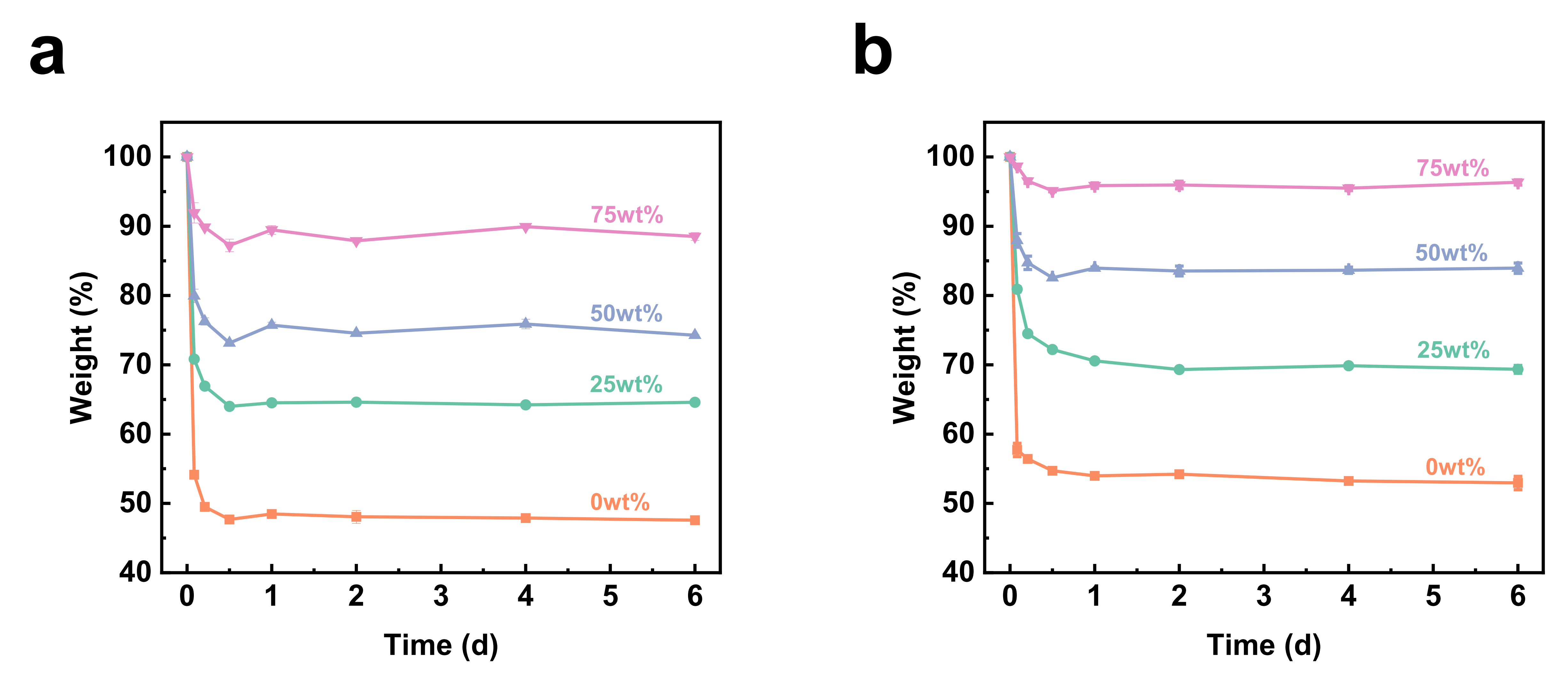


**Figure S8**. Time-dependent weight change of GW-PA-Ag electrodes as a function of glycerol ratio at a temperature of 25℃ and a humidity of 40% and 80%.

**

**

**Figure S9**. Relative cell viability (Rv) of cells cultured with GW-PA, GW-PA-Ag, and control.


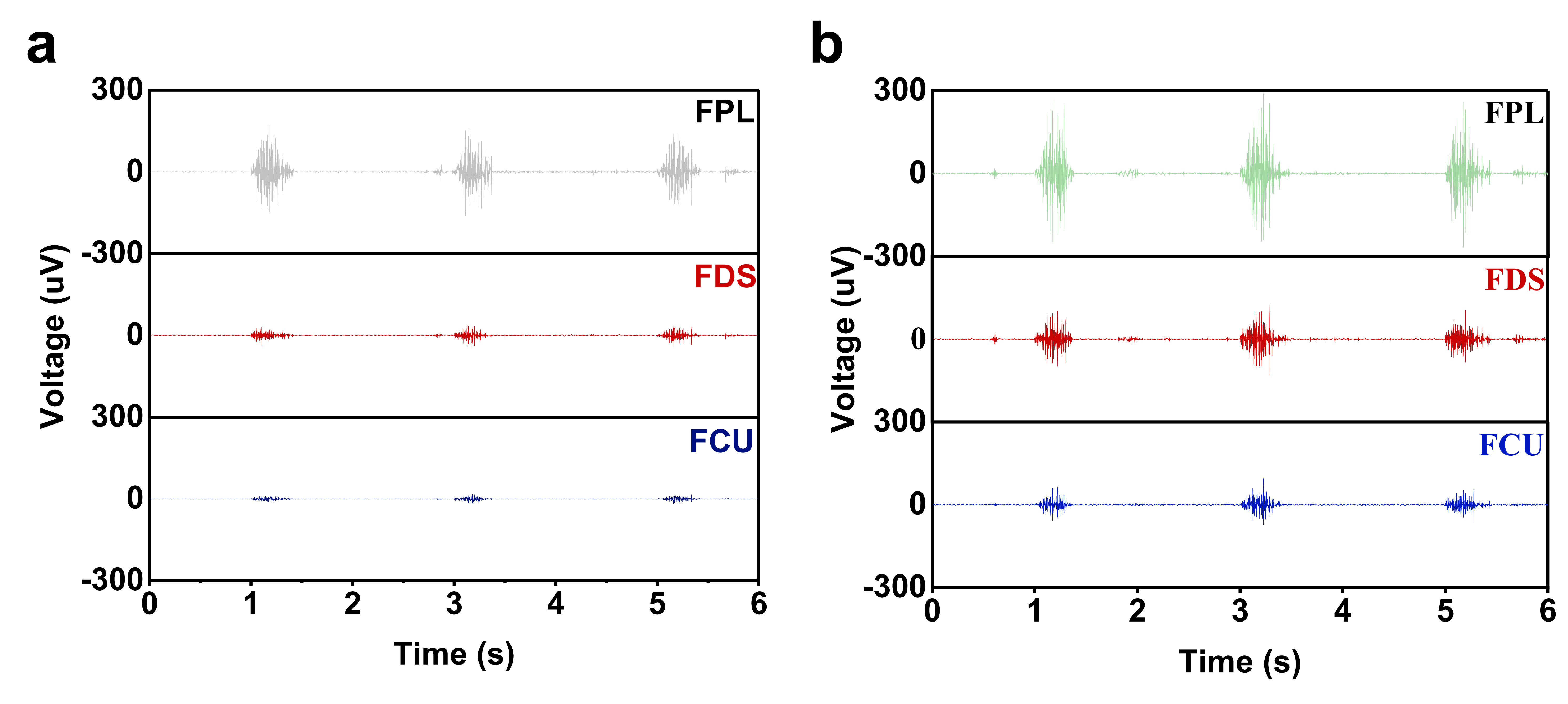


**Figure S10**. sEMG signals’ magnitudes on FPL, FDS, and FCU muscles recorded by a) 3mm, b) 7.5mm diameter electrodes when the thumb finger moves.


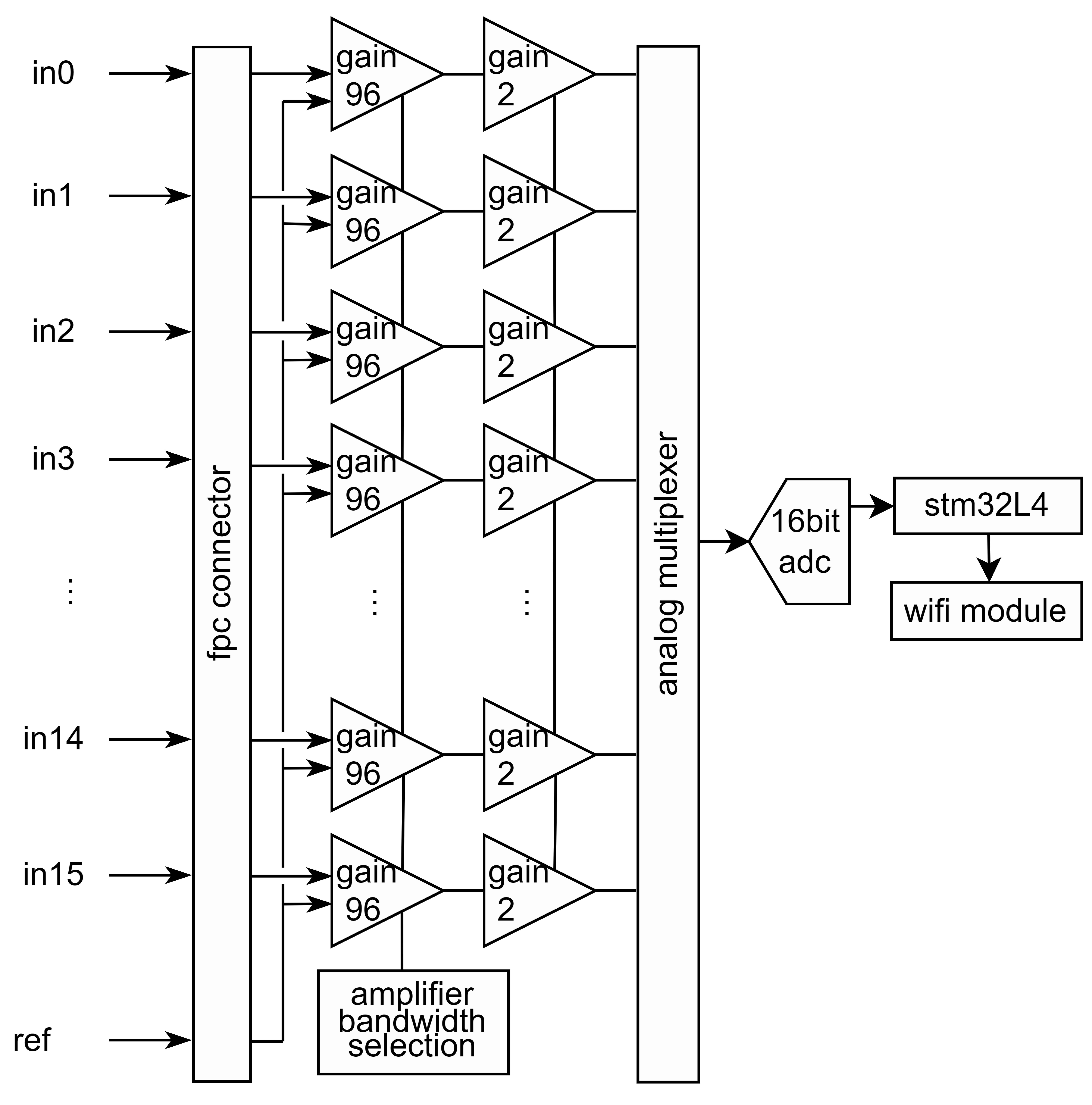


**Figure S11**. Schematic diagram of 16-channel EMG acquisition system.


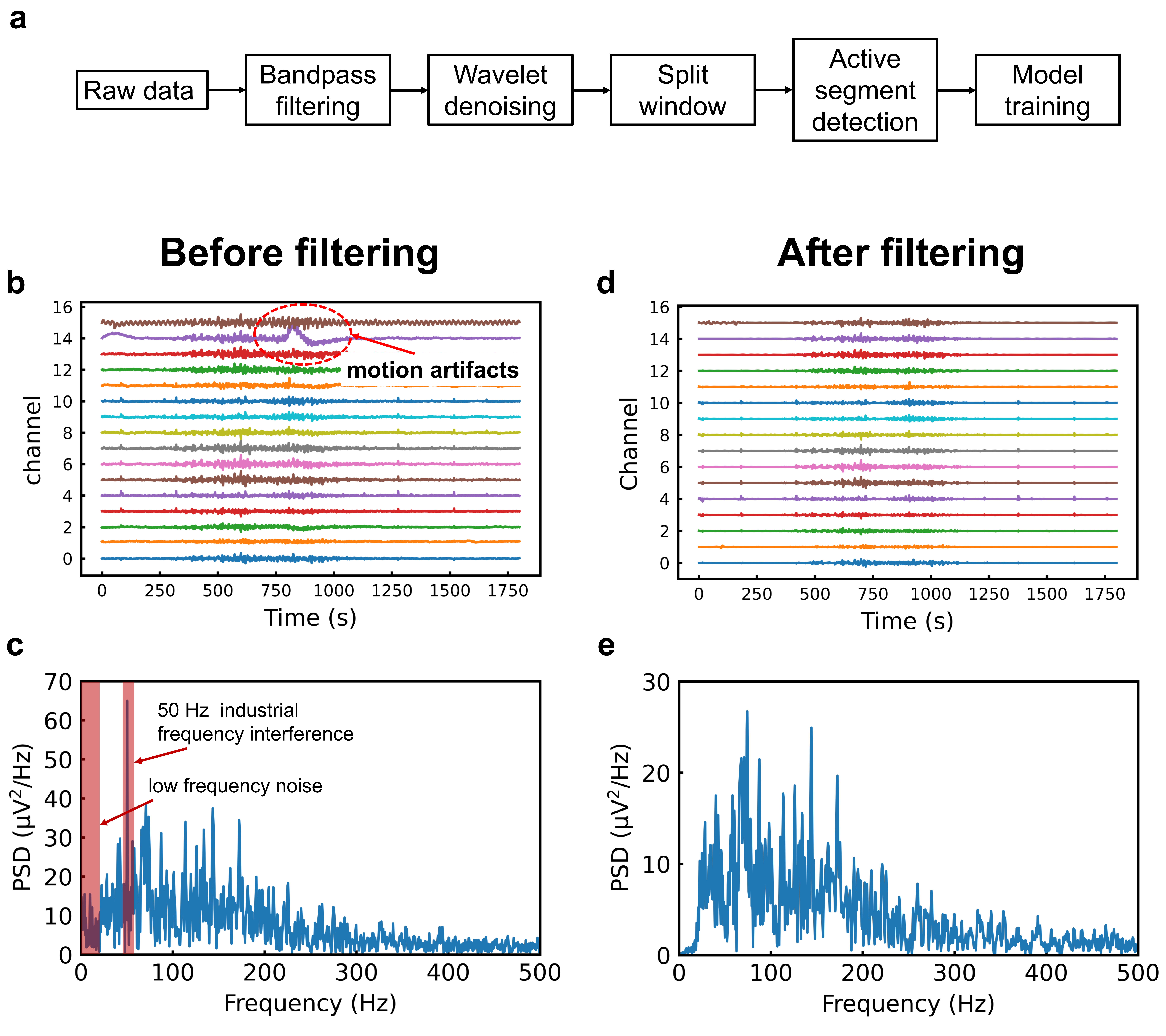


**Figure S12**. a) Data processing flow. b-c) Time and frequency domain plots of the signal before filtering and d-e) after filtering.


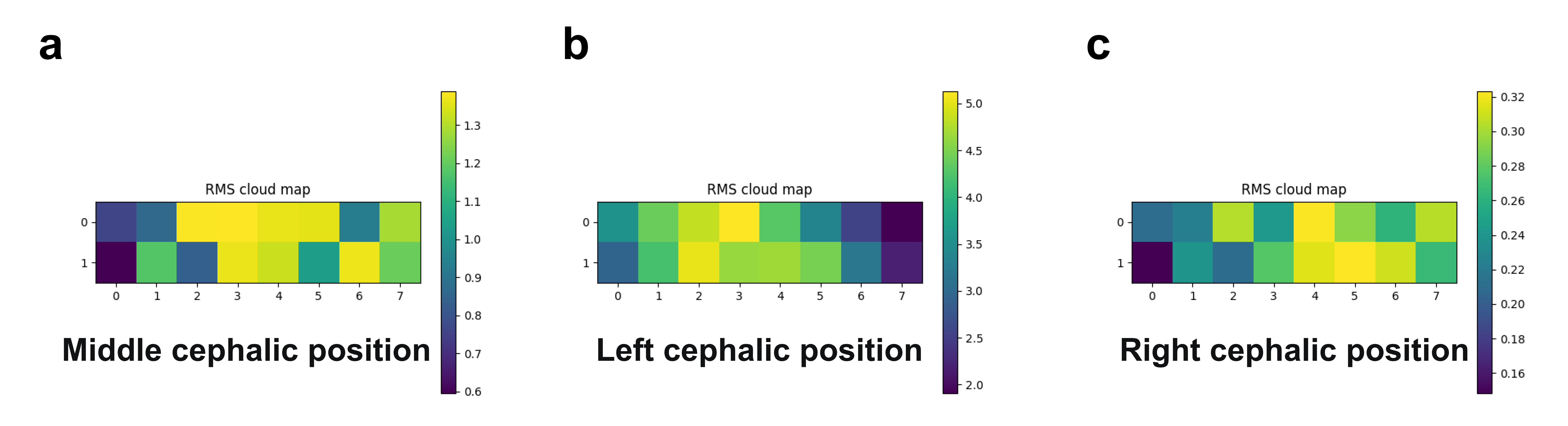


**Figure S13**. sEMG energy maps of swallowing at three head positions.
